# Individualized hydration plans improve performance outcomes for collegiate athletes engaging in in-season training

**DOI:** 10.1186/s12970-018-0230-2

**Published:** 2018-06-04

**Authors:** David Ayotte, Michael P. Corcoran

**Affiliations:** Department of Health Sciences, Merrimack College, O’Reilly Hall, Room 414, 315 Turnpike Street, North Andover, MA 01845 USA

**Keywords:** Sweat testing, Dehydration, Athletes, Prescription hydration plan, Standing long jump, Attention and awareness, Heart rate recovery

## Abstract

**Background:**

Athletes commonly consume insufficient fluid and electrolytes just prior to, or during training and competition. Unlike non-athletes or athletes who do not engage in frequent rigorous and prolonged training sessions, “hard trainers” may require additional sodium and better benefit from a hydration plan tailored to their individual physiology. The purpose of this randomized cross-over study was to determine whether a hydration plan based off of an athlete’s sweat rate and sodium loss improves anaerobic and neurocognitive performance during a moderate to hard training session as well as heart rate recovery from this session.

**Methods:**

Collegiate athletes who were injury free and could exercise at ≥ 75% of their maximum heart rate for a minimum of 45 min were recruited for this randomized, cross-over study. After completing a questionnaire assessing hydration habits, participants were randomized either to a prescription hydration plan (PHP), which considered sweat rate and sodium loss or instructed to follow their normal ad libitum hydration habits (NHP) during training. Attention and awareness, as well as lower body anaerobic power (standing long jump) were assessed immediately before and after a moderate to hard training session of ≥ 45 min. Heart rate recovery was also measured. After a washout period of 7 days, the PHP group repeated the training bout with their normal hydration routine, while the NHP group were provided with a PHP plan and were assessed as previously described.

**Results:**

Fifteen athletes from three different sports, aged 20 ± 0.85 years, participated in this study. Most participants reported feeling somewhat or very dehydrated after a typical training session. Compared to their NHP, participants following a PHP jumped 4.53 ± 3.80 in. farther, tracked moving objects 0.36 ± 0.60 m/second faster, and exhibited a faster heart rate recovery following a moderate to hard training session of 45–120 min in duration.

**Conclusion:**

A tailored hydration plan, based on an athlete’s fluid and sodium loss has the potential to improve anaerobic power, attention and awareness, and heart rate recovery time.

## Background

Suboptimal hydration strategies during training and competition are well known to reduce athletic performance through increased physiological stress [1–6]. Athletes who lose as little as 1–2% of their body mass through sweat loss exhibit an increase in heart rate, core temperature, muscle glycogen use, as well as a decrease in cardiac output, cognitive awareness, anaerobic power, and time to exhaustion [2–6]. Additionally, inadequate replacement of sodium, the predominant electrolyte lost through sweat, is thought to exacerbate the decline of these factors [7]. Hydration beverages that replace both fluid and electrolytes lost through sweat have been employed over the last several decades, as evident with the widely available commercial sports drink market.

However, there is no one universal hydration strategy that athletes can utilize to mitigate dehydration-associated performance declines because each individual sweats at a different rate and loses a unique amount of sodium through this sweat [8]. In a convenience sample of 500 athletes, Baker et al., determined fluid and sodium losses through training to range from 0.3–5.7 L. h^− 1^ and 18.2–70.8 mmol. L^− 1^ (418–1628 mg. L^− 1^) respectively [9]. Based on these numbers, many commercially available sports drinks do not supply enough sodium to replace the amount lost through sweat for many athletes. This prompts the question of whether it is worthwhile to create a hydration plan tailored to the individual athlete or if a more universal strategy is adequate. Compounded with this, is past research, which has shown that athletes seldom have a thorough understanding of what they should be drinking, how much they should be drinking, or how often they should be drinking [10–12]. A study by Torres-McGehee and colleagues found that when 185 athletes were assessed on their knowledge of hydration and intake of micro and macronutrients, only 9% of them exhibited adequate knowledge in these areas of nutrition [12]. A more recent analysis by Abbey et al., showed similar findings when collegiate athletes scored an average of 55% on a nutrition knowledge assessment [10]. Research has also indicated that a majority of athletes have a tendency to rely on a sense of thirst to inform them of when they should be drinking fluids during training sessions and competitions. Unfortunately, when athletes rely on a sense of thirst alone, they do not voluntarily drink enough fluid to prevent the occurrence of dehydration during exercise [8, 11, 13]. This is exacerbated by the fact that a majority of athletes begin training or competition in a somewhat dehydrated state [8, 11, 14]. Overall, the research indicates that the sports performance of many athletes are likely being hindered by substandard hydration habits.

In light of these findings, the purpose of this investigation was to determine whether a prescribed hydration plan that considers both fluid and sodium loss, improves the athletic performance of collegiate athletes engaged in a variety of sports. Here, athletic performance is defined by several metrics: heart rate recovery, anaerobic power, and attention and awareness following a moderate to hard training session of at least 45-min in duration. We also sought to contribute to the findings of Torres-McGehee et al. [12], Abbey et al. [10], and others [15, 16], by determining what collegiate athletes are consuming during training and what their knowledge-base is regarding proper hydration.

## Methods

### Study design

Fifteen collegiate athletes from Merrimack College (NCAA Division I (ice hockey) and II (all other sports)) were recruited for this randomized, crossover study. Participants were eligible if they were between 18 and 24 years of age, injury-free, able to exercise at greater than 75% of their maximal heart rate for a minimum of 45 min, were on one of the college’s sports teams, and provided informed consent. Informed consent was also required by the participant’s head coach and the head athletic trainer on campus. Because the training sessions utilized in this study consisted of already-scheduled team training sessions, athletes were recruited from in-season sports that were currently engaged in heavy sports-specific training sessions. Once recruited, participants underwent a qualitative assessment for hydration habits and knowledge. Participants were interviewed one-on-one by researchers to gauge hydration habits and knowledge pertaining to dehydration and overhydration. This subjective questionnaire consisted of a combination of open-response and multiple-choice questions. The full list of questions are shown in the results section of this study. Following this, participants were assessed for sweat loss, then randomized to either a prescription hydration plan (PHP) or asked to continue with their normal hydration habits (NHP). Participants in each group underwent performance assessments prior, during, and immediately after a moderate to hard training session (goal average heart rate ≥ 75% of maximum for at least 45 min in duration). Maximum heart rate was estimated with the formula, 207-(0.7 x age in years) [17]. Heart rate (HR) was recorded remotely using the Zephyr PSM Training System (Zephyr Technology Corporation, Annapolis, MD, US) [18]. Mean and peak heart rate were recorded throughout the entire training session, including just prior to warm up, warm up, and 15-min cool down. All measurements took place immediately before, during, or after a sports-specific training session. For example, hockey players recruited for this study underwent assessments during a full-pad, on ice, practice. Similarly, Lacrosse players were assessed outdoors on the Lacrosse field during one of the teams harder practice sessions. Athletes were also weighed several times per week in the two weeks preceding the training session for determining fluid loss (see “Sweat Assessment”) as well as prior to the NHP and PHP training sessions in order to determine weight stability.

**Fig. 1:**
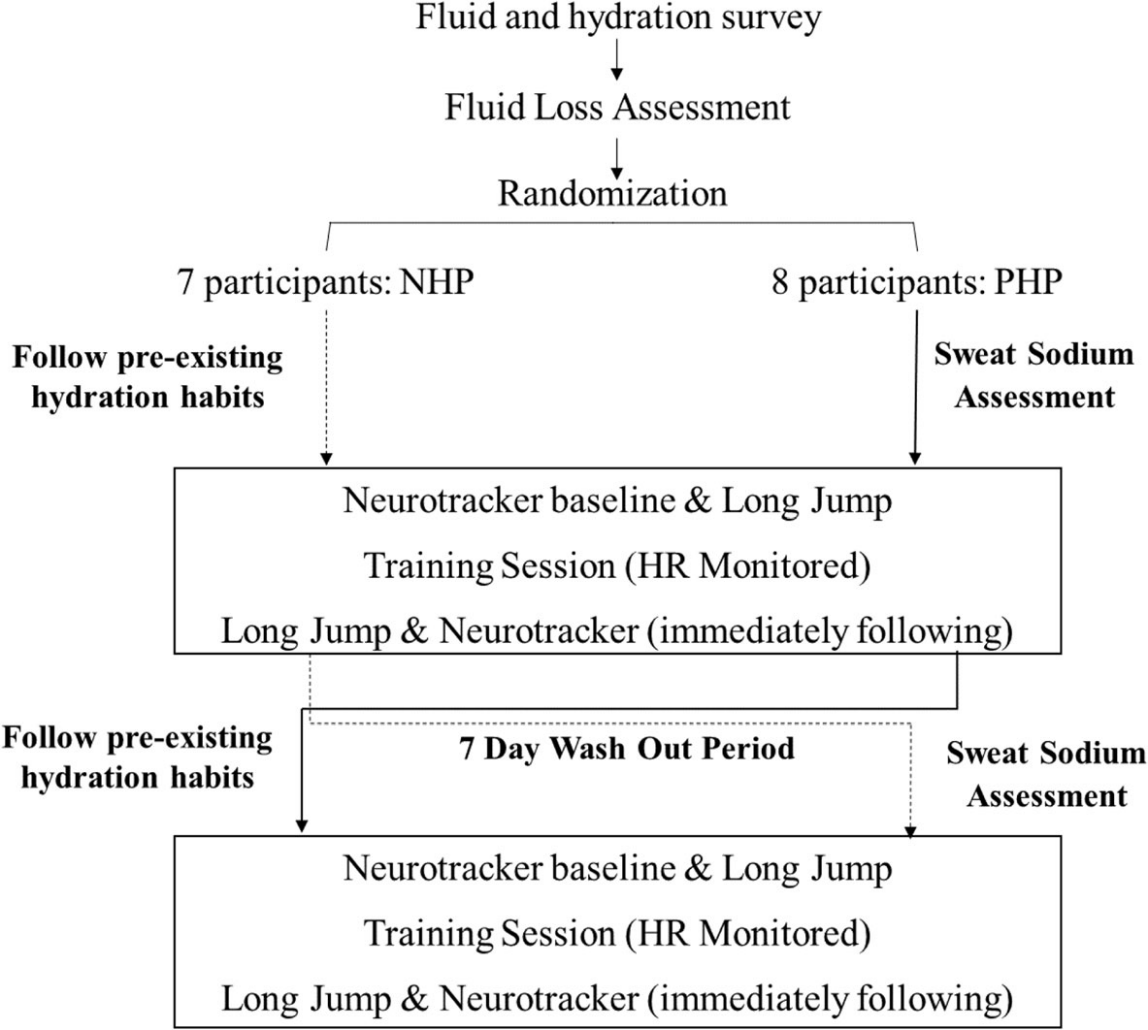
Randomized, cross-over study design to test the effectiveness of a prescription hydration plan on sports performance

The overall design of this study is shown in Fig. 1. Each participant completed a training session with their NHP and PHP, separated by 7 days.

To determine the NHP for each participant, researchers observed the hydration habits of each athlete during at least one training session in addition to reviewing the results of the hydration survey noted earlier. No instruction was provided to athletes with regards to their NHP. Each participant was monitored during their NHP training session for compliance, particularly those who were randomized to follow a PHP first. Researchers also controlled for pre-training hydration status by monitoring fluid consumption beginning at 60 min prior to the start of the sweat assessment, NHP, and PHP training sessions. All fluids consumed during this study were kept at room temperature.

### Sweat assessment

**Fig. 2:**
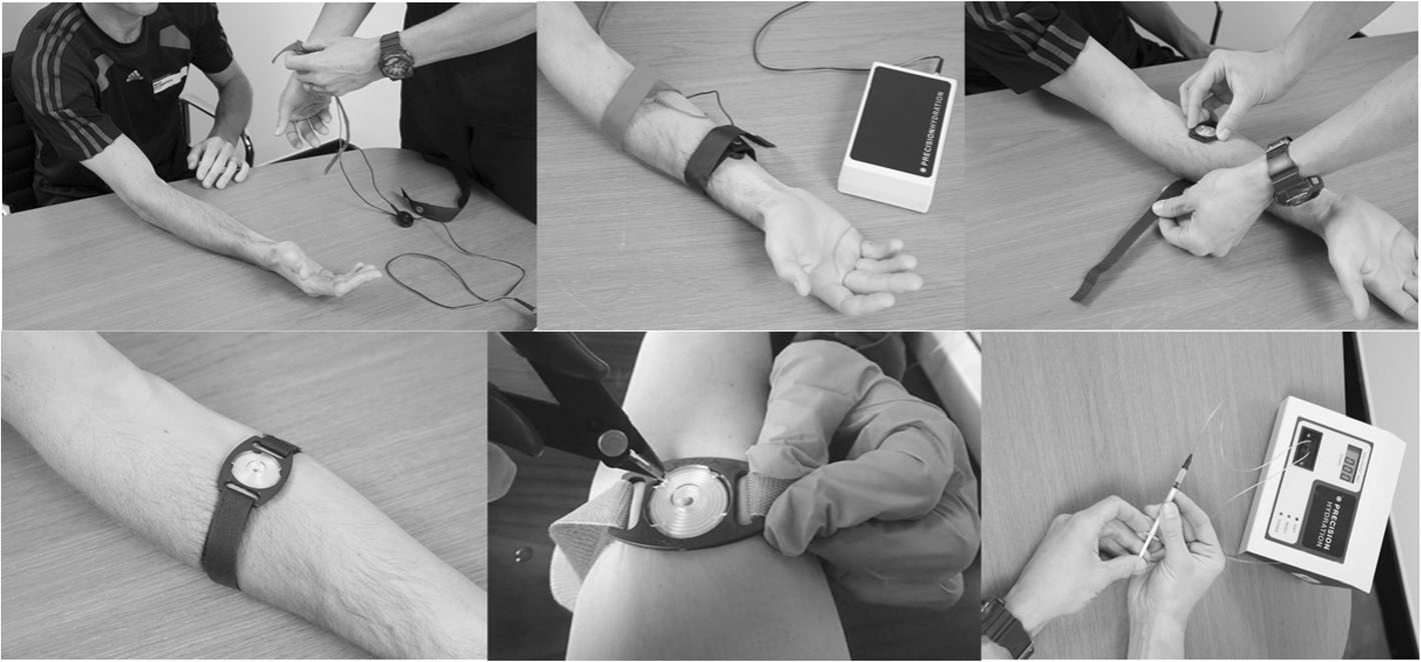
Pilocarpine iontophoresis used for determining the sweat sodium concentration of each athlete

Both fluid loss and sweat sodium (Na^+^) concentration were assessed. Fluid loss from training was performed as described previously [9]. Briefly, nude weight was taken immediately prior to training. Fluid bottles (32 oz of water or sports drink of choice (lemon-lime flavored Gatorade®)) were measured out and provided to each participant. Participants were instructed to only drink from his or her bottle and consumption of fluid was closely monitored during the training session. Participants were again weighed immediately afterwards (nude weight, surface sweat removed via towel dry). The time of day, length of training session, temperature, and level of humidity during the session were also recorded. For reference, all sweat assessments took place during the cooler months (November–March) within the New England region of the U.S. Fluid loss was determined from the change in pre-training to post-training body mass and corrected for fluid intake. Sweat rate was expressed in L. h^− 1^ by taking the total fluid loss and adjusting for the duration of the training session. Relative sweat rate was expressed as ml. kg^− 1^. hr.^− 1^. To determine sweat [Na^+^], sweat was induced and collected from each participant using a Macroduct® Sweat Collection System (ELITech Group, Model #3700 SYS), according to the manufacturer’s instructions [19, 20]. Briefly, sweat induction occurred via the use of Pilogel® Iontophoretic Discs, placed in two electrodes (red and black) that were strapped to the participant’s forearm (Fig. 2). Activation of the sweat inducer served to deliver enough pilocarpine for sweat gland stimulation (equivalent to 5 min of iontophoresis at 1.5 milliamps). Following induction, a macroduct sweat collector was placed over the skin where the red electrode was previously. The collector contained a blue dye that allowed the researchers to observe the collection of sweat by capillary action. Once enough sweat was collected, the [Na^+^] in each sample was assessed using a Sweat•Chek™ Conductivity Analyzer (ELITech Group, Model #3120) as described previously [21].

### Prescription hydration plan (PHP) development

Fluid losses for each athlete (determined previously) were expressed in ounces. 15 min^− 1^. This time measurement was agreed upon by participants and coaches and represented a feasible fluid consumption plan during training sessions. A range of fluid consumption per 15 min was calculated by determining the minimum consumption rate (enough to prevent mild dehydration or 2% bodyweight loss [5]) and the maximum consumption rate (fluid loss determined earlier, which is equated to maintaining, but not exceeding pre-training bodyweight). For example, if an 82 kg athlete with an absolute sweat rate of 1.4 L. h^− 1^ engaged in a 90 min training session, *maximum fluid consumption* was calculated as: 1.4 L × 1.5 h = 2.1 L (71 oz) fluid lost / session. Convert to six 15 min drink intervals in a 90 min session = no more than 11.8 oz. consumed every 15 min. *Minimum fluid consumption* was calculated as follows: 82 kg × 2% = 1.64 kg (57.6 oz. equivalent) allowable sweat loss. 71 oz. lost / session – 57.6 oz. allowed / session = 13.4 oz. (at minimum) that need to be made up via fluid consumption. 13.4 oz. / six 15 min intervals = 2.2 oz. of fluid consumed every 15 min at minimum. This participant would then be advised to consume between 2 oz. to 12 oz. of fluid every 15 min of activity. The bottles used in this study were individually marked for quantity to delineate how much should be consumed at each 15 min interval. More specifically, there would be two markings for each 15 min interval such as “Min-15”, “Max-15”, “Min-30”, and “Max-30,” beginning from the top of the bottle to the bottom. The exact volumes would vary from athlete to athlete and each participant would be instructed to sip their bottle at each interval such that the fluid line was between the minimum and maximum. For athletes engaging in training sessions that exceeded the fluid capacity of the bottle, multiple similarly marked bottles would be provided. Researchers monitored fluid consumption throughout the training session to gauge whether an athlete was on track with their prescribed volume. The composition of fluid that each participant consumed for the PHP was based upon what he or she regularly consumed (NHP fluid), supplemented with a level of NaCl corresponding to the participant’s sweat sodium loss. This usually involved adding NaCl to 32 oz. of a commercially available sports drink or water depending upon which beverage-type was normally consumed by the individual. For example, if an athlete lost 43.5 mmol Na^+^. L^− 1^ (1000 mg Na^+^. L^− 1^) of sweat and preferred lemon-lime Gatorade G2™, which contains 480 mg sodium/32 oz [22], the researchers would add 520 mg NaCl to the beverage to create a solution that was isotonic relative to sweat sodium content. Lastly, 30 min prior to engaging in a PHP training session, participants were instructed to consume 8 oz of their prescribed beverage.

### Neurotracker

Spatial awareness and attention was assessed by 3-dimensional multiple object tracking (3D-MOT) capacity via the NeuroTracker™ system (CogniSens Athletic Inc., Montreal, Canada) as described previously [23, 24]. All testing was conducted in a quiet, dimly lit room with minimal outside distractions and consisted of three 10 min trials interspersed with five minute rest periods. During these assessments, participants wore 3D glasses and were required to track designated objects on a screen as they moved in variable patterns and at subsequently faster speeds. Each of the assessments began at a preliminary speed of 1.0 m per second. The degree of difficulty associated with the assessment progressively increased with every correct answer provided by the participants. In contrast, the level of difficulty associated with the assessment progressively decreased with every incorrect answer. The mean score of the three trials (expressed as tracking speed in meters/second) was used. Each participant performed the neurotracker assessments before and immediately after the training sessions. Changes in spatial awareness and attention were illustrated by comparing pre-training with post-training scores.

### Standing long jump

To gauge lower body anaerobic power [25], three standing long jump tests (SLJs) were performed before and after the NHP or PHP training sessions. The pre-training SLJs immediately followed the neurotracker assessments, while the post-training SLJs preceded the neurotracker. Prior to completing the first of the three maximal SLJs, each participant completed two submaximal trials to become familiarized with the protocol. For the test itself, participants were instructed to stand with their feet should-width apart behind a starting line. On the command “ready, set, jump!” the participants executed the jump. Researchers measured each of the jumps from the participants’ rear-most heel and took the average of the three attempts in inches.

### Statistical analysis

Wilcoxon Signed Rank test for paired samples was conducted in order to determine if there was a significant difference in the pre and post athletic performance measurements and when participants followed their normal hydration plans compared to when they followed their individualized prescription hydration plans. All data are presented as means ± SD except where otherwise specified. SPSS 23 for Windows (IBM SPSS, Chicago, IL) was used for all statistical analyses. GraphPad Prism® software (version 6.07) was used for graphical displays. A value of *P* <  0.05 was regarded as statistically significant. Where statistically significant effects were observed, effect sizes (Cohen’s D) were determined by assessing the differences between the two group means based on > 0.2 SD indicating a small effect, > 0.5 a moderate effect, and > 0.8, a large effect [26].

## Results

Fifteen NCAA Division I and II athletes from three different sports participated in this study. Participant demographics are shown in Table 1. Relative and absolute sweat rates were 1.3 ± 0.6 L•hr.^− 1^ and 18.8 ± 7.5 mL•kg^− 1^•hr.^− 1^ respectively while sodium loss was 24.6 ± 7.1 mmol•L^− 1^. The training sessions on average lasted 90 min (range of 45–120 min) with participants exerting themselves at 78–79% of their maximum heart rate. Seven of the 15 participants engaged in 120-min training sessions, 6 engaged in 70 min sessions, and 2 engaged in 65 min and 45-min training sessions respectively. The duration and structure of the NHP and PHP training sessions did not differ for each participant. All participants had practice in the afternoon or evening. The time of day of the NHP and PHP sessions did not differ among any of the athletes in this study.

**Table 1:** Baseline characteristics and exercise training of participants

	All	NHP	PHP
Participants (N)	15		
Female	9		
Male	6		
Age (years)	20 ± 0.85		
Weight (kg)	70.9 ± 9.1		
Sport			
Women’s Ice Hockey	6		
Women’s Lacrosse	3		
Men’s Lacrosse	3		
Men’s Track & Field	3		
Sweat Assessment			
Absolute sweat rate (L•hr.^−1^)	1.3 ± 0.6		
Relative sweat rate (mL•kg^−1^•hr.^− 1^)	18.8 ± 7.5		
Sodium Loss (mmol•L^− 1^)	24.6 ± 7.1		
Training Conditions^a^			
Temperature (°C)	7.9 ± 4.3		
Relative Humidity (%)	37 ± 4.0		
Training Bout			
Training Duration (mins)	91.2 ± 28.1	91.3 ± 28.4	91.0 ± 28.8
Training Heart Rate (bpm)^b^	152 ± 6	153 ± 6	151 ± 6
% of Max HR	79 ± 3	79 ± 3	78 ± 3

Numbers expressed are means ±SD, unless otherwise stated

^a^Training conditions did not significantly differ between sweat assessment session and NHP or PHP training sessions

^b^Does not include warm-up or cool down period

The results of the fluid and hydration survey, including the normal hydration habits of the participants in this study are shown in Table 2. Sixty percent of the participants in this study believed that their current hydration strategies were effective despite 40% reporting that they feel very dehydrated during a training session. Most participants consumed water during training, as it was usually the only fluid available.

**Table 2:** Fluid and hydration survey results

Question	Response (n)
How often do you consider hydration before practice?	Consider very much (6)
	Consider somewhat (7)
	Consider not often (2)
How often do you consume fluids during practices/competitions?	Often / Every 15–30 min (7)
	Somewhat Often / Every 30–60 min (6)
	Rarely to Never (2)
What type of hydration beverages do you normally consume during practices/competitions?	Water (12)
	Sports Drink (3)
How much fluid do you usually consume during practices/competitions?	More than 12 oz (2)
	Less than 12 oz (5)
	Less than 8 oz (6)
	Not Sure (2)
Do you believe that your current hydration strategies are effective?	Yes (9)
	No (6)
How dehydrated do you feel during practices/competitions?	Very dehydrated (6)
	Somewhat dehydrated (7)
	Not dehydrated (2)
How often do you consider hydration after practice?	Consider very much (9)
	Consider somewhat (5)
	Consider not often (1)
Where did you learn your current hydration strategies from?	Parents (8)
	Coaches (6)
	Athletic Trainers (5)
	Nutritionist (5)
	Teammates (4)
	Nutrition Class (3)
	Other (3)
Do you believe that it is possible to overhydrate?	Yes (11)
	No (4)
Do you believe that overhydration improves or impairs athletic performance?	Impaires (13)
	Improves (2)
Do you believe that thirst alone can be a predictor of dehydration?	Strongly agree (1)
	Agree (6)
	Neutral (3)
	Disagree (3)
	Strongly disagree (2)

### Within group differences (training effect)

**Fig. 3:**
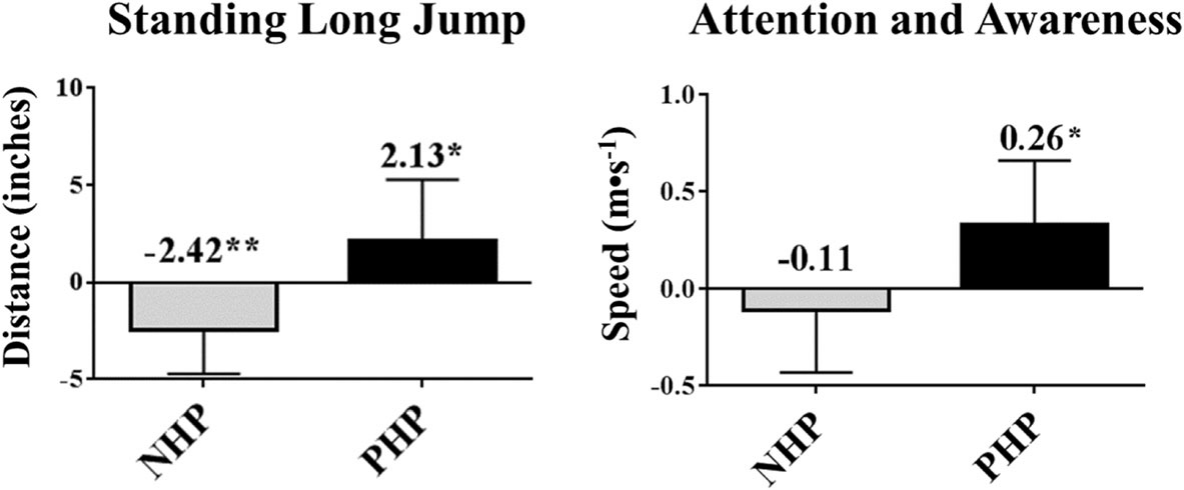
Change in performance following a 45–120 min bout of moderate to hard training. * = *P* <  0.05, ** = *P* < 0.01

All participants in the study complied with their respective prescription hydration plans. Compared with pre-training performance, participants jumped 2.42 ± 2.29 in. shorter after training when following their NHP (Fig. 3). In contrast, when these participants followed a PHP, they jumped 2.13 ± 3.15 in. farther post-training compared with pre-training performance. Similarly, attention and awareness improved when participants followed a prescription hydration plan. After training with their NHP, participants on average experienced a non-significant reduction of 0.11 ± 0.32 m•s^− 1^ in their ability to track moving objects compared with pre-training tracking speed (Fig. 3). In contrast, when following their PHP, participants significantly improved object tracking ability by 0.26 ± 0.40 m•s^− 1^.

### Between group differences (PHP effect)

**Fig. 4:**
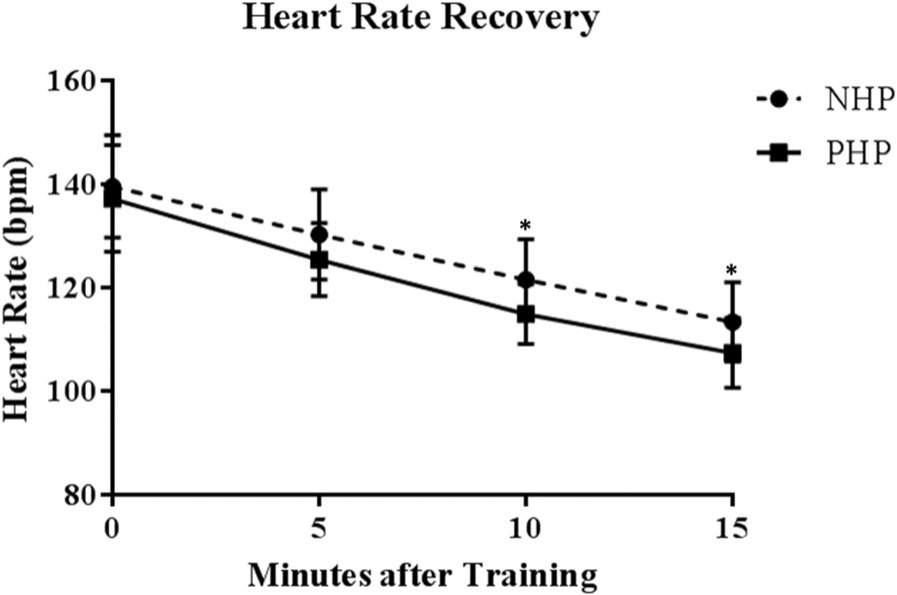
Heart rate recovery after completing a training session with each hydration plan

Heart rate recovery was faster post-training when participants followed a PHP as compared with their respective normal hydration plans (Fig. 4). These differences were significant at 10 min and 15 min post-training (Table 3). Similarly, standing long jump performance as well as attention and awareness was also improved. The effect size for both heart rate recovery (− 4.47 ± 6.71 bpm at 10 min, − 3.73 ± 6.58 bpm at 15 min) and standing long jump performance (4.53 ± 3.80 in.) was large.

**Table 3:** Effect of a prescription hydration plan on performance relative to an ad libitum hydration plan

Variable	Difference btw means	95% CI	*p*-value	Effect size (Cohen’s D)
Standing Long Jump (in)	4.53 ± 3.80	2.43 – 6.64	< 0.0001	Large
Attention and Awareness (m/s)	0.36 ± 0.60	0.03 – 0.70	0.0302	Small
HR_Rec_ (5 min post) (bpm)	−2.60 ± 6.82	−6.38 – 1.18	0.1838	NS
HR_Rec_ (10 min post) (bpm)	−4.47 ± 6.71	−8.18 – −0.75	0.0087	Large
HR_Rec_ (15 min post) (bpm)	−3.73 ± 6.58	−7.38 – −0.09	0.0139	Large

Differences are means ± SD

## Discussion

This study investigated whether an individually tailored hydration plan improves performance outcomes for collegiate athletes engaged in seasonal sports. Participants were recruited from three sports (ice hockey, lacrosse, and track & field) as these sports were currently in season and the athletes were engaged in consistent and standardized training sessions. All athletes in this study had practice in the afternoon or evening with the NHP and PHP sessions occurring at the same time of day for each individual. A prescription hydration plan (PHP) was created for each participant that was based on both fluid and sodium losses incurred during moderate to hard training sessions lasting at least 45 min in duration. Athletes were instructed to drink at 15 min intervals at a volume of fluid that prevents a 2% bodyweight loss as well as any weight gain. A maximum fluid consumption level for each PHP was established as a precaution, given that overhydration is a well-known risk factor for exercise-induced hyponatremia [27]. However, the likelihood of this occurring in this study was low given that the athlete cohort in this study engaged in training sessions lasting no more than 120 min [28]. The fluid itself was isotonic relative to the athlete’s specific sweat sodium concentration and was based off of fluids readily available to him or her. The results indicate that this approach was effective in improving heart rate recovery, attention and awareness, and mitigating the loss in anaerobic power that occurred from the training session. Compliance was high with the prescribed volume of fluid well tolerated by the participants. While some athletes did remark that they could taste the extra sodium, this did not appear to affect the compliance to their prescribed hydration protocol, even among those who required the most salt added to their beverage.

To our knowledge, this is the first investigation to look at whether an individually tailored hydration plan improves athletic performance for collegiate athletes engaged in a variety of sports. Previous work has shown that hydration plans based purely on fluid loss hold promise [13]. Bardis et al., examined whether consuming water at regular intervals to offset fluid losses as compared with ad libitum fluid consumption improved the performance of cyclists [13]. The researchers found that power output was maintained throughout a training session consisting of three 5-km hill repeats, whereas when these cyclists consumed water ad libitum, their power output dropped with each successive repeat [13]. Other studies have examined the effects of isotonic beverages on sports performance, yet often compare such beverages to water [29–31]. This presents the obvious issue of accounting for any carbohydrate effect as most commercially available sports drinks are 6–10% carbohydrate solutions mixed with several electrolytes, among them sodium. In this study, because the specific beverage consumed by each participant was held consistent between the NHP and PHP training sessions, the results are not confounded by factors such as the carbohydrate composition of a beverage. The PHP intervention manipulated only the fluid quantity and sodium consumed immediately before and during exercise.

In all cases, the final [Na^+^] of the PHP beverages were higher than any of the sports drinks available to our athletes (though most habitually consumed water during training). With the notable exception of endurance-focused sports drinks, many commercially available beverages do not match the sodium loss rate of many individuals. This is understandable as it is commercially untenable to create a sports drink unique to every individual’s sweat composition. For the majority of individuals engaged in recreational physical activity these drinks are more than sufficient. For elite and amateur athletes looking for every possible safe method to improve performance, the results of this study support commercial sweat testing in order to develop optimal hydration strategies. This may hold especially true for athletes engaged in longer sporting events such as a marathon or Ironman triathlon, where the loss of fluid through sweat is substantial [32]. Supplementation with higher sodium sports drinks or salt capsules may be advisable for athletes engaged in prolonged exercise of 3 h or more in order to maintain serum electrolyte concentrations [33, 34]. Based on these studies and others, the longer an event, the more critical it appears to be to have an adequate hydration plan in place that considers sweat rate and composition [1, 34]. In our study, most of the participants engaged in training sessions lasting between 70 min to two hours and the benefits were apparent.

Lastly, in line with previous work, we also found that while most athletes in this study felt that their current hydration strategies were effective, the majority of this cohort reported feeling dehydrated after a training session [10, 11, 15, 16]. The disconnect between ad libitum fluid consumption and hydration status during competition is well documented [8, 11, 13, 15]. Studies have consistently shown that it is not uncommon for athletes to show up to a training session already dehydrated and consume inadequate fluid levels despite the ready availability of water or sports drinks [8, 11, 14–16]. It cannot be definitively stated whether the athletes in our study were dehydrated at the beginning of practice. In this study, the researchers were present to monitor compliance to the prescribed fluid volume, including the pre-practice consumption of the PHP beverage. While the PHP used in the present work was feasible to create and implement, ensuring compliance in day to day training may be challenging. In a study by Logan-Sprenger et al., a third of all ice hockey players failed to hydrate adequately during a game despite these fluids being readily available [15]. Increasing hydration awareness along with providing pre-marked bottles that state how much fluid should be consumed by set time periods, if feasible, may be one approach to overcoming this issue.

### Study limitations

This study has several limitations. First, only one training session was utilized per hydration plan. Based on researcher observations, participant feedback, and input by coaches, there was little difference in the training sessions used for the NHP and PHP assessments with each participant. It was important to control for the training sessions utilized as well as ensuring minimal fitness gains in between NHP and PHP sessions. Hence, we required a “wash out” period of 7 days. The training sessions utilized in this study were already pre-scheduled so as not to interfere with the practice plan that each coach designed for their athletes. For each sport at the college where and when this study occurred, the number of ideal sessions to test the PHP were limited. The fact that multiple sports were used to test the PHP is both a strength (broad applicability) and a limitation (non-specific). Given the team schedules and the timing of this study during the winter/spring seasons in the New England, USA area, it is unclear what affect a warmer, more humid climate may have had on the results. Given that both the NHP and PHP training sessions were similar in duration, intensity, mode of training, and climate, we postulate that these results will hold in warmer conditions. More so, given higher degrees of fluid loss with warmer, more humid climates, the benefits from the PHP observed in this study may even be amplified to a certain degree. This is speculative however and future studies if feasible, should consider testing athletes over multiple training sessions per treatment. Additionally, in this study, sweat sodium concentrations were assessed at the forearm. Previous research has indicated that measuring sodium from multiple body sites such as was done by Dziedzic et al., can lead to higher sweat sodium values [35]. Based on Dziedzic’s report, we determined that this difference translates to adding roughly 200 mg more sodium per a 32-oz sports beverage than what was added to the 32 oz beverages in this study. We are unclear on what impact this additional salt may have made concerning the performance outcomes used in this study. From a practical standpoint, assessing the forearm is often a more feasible approach to determining sweat sodium concentrations than a whole-body approach. Another limitation to this study is that it relied on bodyweight changes and fluid intake monitoring to gauge hydration status. This method is less precise than other methods of hydration status such as a urine specific gravity test (USG) [36]. We were unable to conduct a USG due to equipment limitations. We did note however, the bodyweight trends of all athletes in this study over the two weeks preceding the pre-training bodyweight measurements (data not shown). There was no significant difference in these weights as compared with the pre-training bodyweights (taken during the NHP and/or PHP training sessions), indicating that each athlete was weight-stable. This however does not negate the possibility that an athlete was dehydrated, euhydrated or hyperhydrated going into each training session. Further research should include tests such as USG so that hydration status can be confidently determined.

There are also several potential confounders that need to be addressed. Factors such as sleep quality, personal stress, medication use, menstrual cycle, and diet may have affected the outcomes. We did not assess nor control for the athlete’s environment outside of one hour from the training session. One main advantage of the randomized, cross-over design utilized for this study is that each participant served as his or her own control, which presumably minimized the influence of any potential confounding covariates. Despite the strength of this design, future studies in hydration research may do well to assess diet, stress level, and sleep quality as mentally, these factors can significantly impact athletic performance. Collegiate athletes are not immune to the stresses of balancing both academic and athletic responsibilities in addition to managing personal stressors common to all segments of the population.

## Conclusions

In summary, this investigation showed that a hydration plan based on an individual’s sweat rate and sodium loss has the potential to markedly improve athletic performance for collegiate athletes engaging in seasonal sports. The understanding that athletes sweat and lose electrolytes at a variety of different rates is something that professionals in the exercise science and sport nutrition fields need to be aware of in terms of optimizing their athletes’ health, safety and performance. While requiring additional effort upon the team staff, determining hydration plans for each athlete is a simple, safe, and effective strategy to enable athletes to perform at their current potential. Future studies should continue in this area and build upon the findings of this report.
